# Immunopathological syndromes: state of the art

**DOI:** 10.3389/fmed.2025.1633624

**Published:** 2025-08-13

**Authors:** Dmitry Kudlay, Vladimir Kozlov, Andrey A. Savchenko, Andrey Simbirtsev, Evgenia Anisimova, Igor Kudryavtsev, Anastasia Kulpina, Artem Rubinstein, Varvara A. Ryabkova, Leonid P. Churilov, Olga Sirotkina, Tatyana Vavilova, Anna An. Starshinova, Alexandr Borisov

**Affiliations:** ^1^I.M. Sechenov First Moscow State Medical University, Institute of Pharmacy, Department of Pharmacology, Moscow, Russia; ^2^Institute of Immunology FMBA of Russia, Laboratory of Personalized Medicine and Molecular Immunology, Moscow, Russia; ^3^Lomonosov Moscow State University, Department of Pharmacognosy and Industrial Pharmacy, Faculty of Fundamental Medicine, Moscow, Russia; ^4^Federal Research Center “Krasnoyarsk Science Center” of the Siberian Branch of the Russian Academy of Sciences, Scientific Research Institute of Medical Problems of the North, Krasnoyarsk, Russia; ^5^State Research Institute of Highly Pure Biopreparations, Saint Petersburg, Russia; ^6^Institution of Experimental Medicine, Department of Immunology, St. Petersburg, Russia; ^7^Almazov National Medical Research Centre, Medical Department, Saint-Petersburg, Russia; ^8^Saint Petersburg State University, Department of Mathematics and Computer Science and Medical Department, St. Petersburg, Russia; ^9^Saint Petersburg State University, the Laboratory of the Mosaic of Autoimmunity, St. Petersburg, Russia; ^10^Cardiological Department at the Clinical Hospital of I.P. Pavlov 1st Saint Petersburg State Medical University, St. Petersburg, Russia

**Keywords:** adaptive immunity, immune system, immunothrombosis, immune pathology, COVID-19, diagnostics of immune disorders

## Abstract

The review of the current state of knowledge on local and systemic immunopathological reactions of cellular and humoral origin, as well as the ways of their interaction, is considered in this article. This study aimed to organize, standardize, and conceptualize existing knowledge about immunopathological syndromes associated with innate immunity. It highlights syndromes linked to type I, II, and III hypersensitivity reactions, while also separately examining manifestations related to immunosuppression disorders. The review outlines how to differentiate humoral immunity syndromes based on the classes of immunoglobulins A, M, E, and the four subclasses of immunoglobulin G. Additionally, it provides a detailed analysis of complement system disorders and the mechanisms of systemic inflammatory response syndrome, as well as their role in various pathological processes. The authors advocate for a unified set of definitions for immunopathological syndromes related to adaptive immunity, aiming to develop a new concept of their pathogenesis. Currently, many definitions of these syndromes lack consensus, stemming from varying interpretations of their manifestations. The authors also propose standardized tools for assessing immunopathological syndromes, along with guidelines for staging and treatment optimization.

## 1 Introduction

Innate immunity acts as the body's first line of defense against pathogens and other injuries. It is activated almost immediately by pathogen-associated molecular patterns and/or products of cell destruction (danger-associated molecular patterns or alarmins). Innate immunity plays a crucial role in the inflammatory process, localizing infections and providing protective and healing effects. Autacoids released by innate immunity cells not only stimulate local inflammation but also trigger systemic inflammatory responses of varying intensities, such as the acute phase response, leukocytosis, and stress reactions that often occur simultaneously. However, all defense mechanisms come with a cost: excessive systemic action of inflammatory mediators can lead to significant pathology (1, 2).

The most important stage in diagnosing immunopathological entities is the detection of local (focal) signs of inflammation and its systemic correlates. The primary clinical syndrome of local inflammation is well-known to physicians of any specialty. The basic signs of inflammation have been described since the time of Aulus Cornelius Celsus (c. 25 BC–c. 50 AD). Galen (129–216) described local heat, swelling, pain, redness, and organ dysfunction, which was later expanded in the 19th century by Rudolf Virchow (1821–1902) (3, 4).

The primary objective of localized inflammation is to isolate and eliminate the phlogogen, followed by repairing the damaged tissue and restoring the impaired barrier function of the body integument. The loss of the barrier function may result from direct action of the pathogens, as well as from primary integument defects, including a decrease in the expression of antimicrobial factors. Irritation of the non-myelinated type C and thin myelinated type Aδ nerve fibers, which contribute to the development of itching, may also be associated with barrier loss. Similarly, dysbacteriosis occurring at the site of inflammation is associated with the development of this syndrome (5, 6).

There are over 450 types of primary inherited immunodeficiencies, as well as numerous secondary acquired immunopathological conditions. Each type has unique diagnostic characteristics and necessitates a tailored treatment strategy. Diagnosing immunopathological syndromes poses a considerable challenge for healthcare providers due to their wide variety, the complexities of laboratory verification, and the overlap of their symptoms with those of other diseases (7).

Given that the immune system is one of the main regulatory and homeostatic systems of the body, it is directly or indirectly involved in the formation of all diseases. However, several syndromes are primarily formed by the immune system itself. Currently, the definitions of some immunopathological syndromes are not universally accepted. There is a disagreement on the interpretation of the manifestations of a particular syndrome. For example, systemic inflammatory response syndrome (SIRS) can be easily defined; however, the boundary between the typical acute phase response accompanying any infection and hyperergic, unregulated response to infection is blurred. The clinician needs to recognize such dysregulation in time, as it has both prognostic and therapeutic implications.

This review aims to systematize, unify, and conceptualize current knowledge on the immunopathological syndromes associated with innate immunity.

## 2 Adaptive immunity

Adaptive immunity is a specific response of the immune system to antigens that develops throughout a person's life, leaving a clear immunological memory, and is provided by specialized cells of great clonal diversity and molecules capable of precisely recognizing and neutralizing specific antigens. Adaptive immunity plays a key role in protecting the organism from various genetically alien cells and molecules (microorganisms, mutated cells, exo- and endotoxins, etc.). It is the basis for the action of vaccines that “train” the immune system to recognize pathogens before their direct contact (1, 4–6).

The presentation of antigens by specialized cells, including dendritic cells, veiled cells, Langerhans cells, as well as macrophages and clonally restricted B-lymphocytes, plays a critical role in activating adaptive immunity. T-lymphocytes, comprising cytotoxic T cells and T-helper cells, are responsible for cellular responses, whereas plasma cells derived from B-lymphocytes contribute to humoral immunity through the production of antibodies (immunoglobulins). The results of these interactions lead to local inflammation, which also involves innate immunity mechanisms. After the initial exposure to an antigen, adaptive immunity establishes immunological memory, enabling a quicker and more effective initiation of a specific immune response in the future.

Several immunopathological syndromes associated with cellular and humoral aspects of the immune response are currently distinguished, based on their pathogenesis (1, 7). The cellular immune response to a pathogen/antigen, according to current views, is defined and detailed as T1-, T2-, and T3-dependent responses (Table 1).

**Table 1:** Immunopathological responses are associated with adaptive immunity reactions (cellular response).

**Response type**	**Immune response factors**	**Pathological entities**	
		**Hyporeactive**	**Hyperreactive**
1st type	ILC1, CD4^+^ Th1, plasmacytoid cells (pDCs), and classical dendritic cells (cDCs1). Cytokines, IL-12, IL-18, IL-15, IFNγ, TNFα, Effector cells, NK- and CD8+ T-cells, and tissue macrophages	Viral infections Intracellular bacterial infections Oncopathology	EAACI reaction type IVa
2nd type	Classical dendritic cells (cDC2), ILC2, CD4+Th2, cytokines, TSLP, IL-25, IL-33, IL-4, IL-5, IL-9 and IL-13. Effector cells, eosinophils, mast cells, basophils, and macrophages	Intestinal main infections	EAACI reaction type IVb
3rd type	cDC2, ILC3, CD4+ Th17 Cytokines, IL-17, IL-22, GM-CSF, IL-6, IL-23 IL-1β, IL-12, IL-27, IL-35, and IL-39 Effector cells, monocytes, macrophages, granulocytes	Bacterial infections	EAACI reaction type IVc
Immunosuppressive response	Treg, Breg, myeloid suppressor cells; anti-idiotypic antibodies	Autoimmune diseases	Infections, oncopathology

*T2-response reactions* (EAACI IVb hyperreactions) are mediated by Th2 cells, which acquire their phenotype under the influence of IL-4. Th2 cells produce IL-4, IL-5, IL-9, IL-13, IL-31, and eotaxins (CCL11, CCL24, and CCL26).

IL-4 and IL-13 are key cytokines of the Th2 response. They switch B-lymphocytes from synthesizing class IgM and IgG_1_ to IgE production. IL-13 is also responsible for tissue remodeling. IL-5 promotes the growth of eosinophils in the bone marrow, the recruitment of eosinophils to sites of inflammation, and their survival in tissues. IL-31 is a major cytokine that plays a mechanistic role in pruritus. Eotaxins belong to the CC subfamily of chemokines, serving as chemoattractants for eosinophils and promoting inflammation; some of them also produce anti-inflammatory mediators. When exposed to IL-4 and TGF-β, Th9 cells differentiate and produce IL-9, which increases IgE synthesis, a growth factor for eosinophil and basophil bone marrow precursors, preventing their apoptosis (5, 8–10).

Lymphoid cells of type 2 innate immunity (ILC2) produce type 2 cytokines (IL-5, IL-13, IL-9, and amphiregulin (a protein that promotes epithelial cell growth), forming a T2 immune response against helminths, inducing tissue inflammation, and maintaining tissue homeostasis. Macrophages, basophils, and ILC2 provide an early source of IL-4, which is involved in Th2 cell differentiation. In addition, IL-4 is produced by a unique subset of invariant natural killer (iNK-T) cells, which contribute to the activation of CD4+ and CD8+ Th2 cells, as well as the initiation and continuation of Th2 inflammation through the production of IL-4. In addition, a small fraction of NK- and NKT-cells produce IL-13. IL-4 and IL-13 induce an alternative activation program in macrophages, resulting in M2 phenotype macrophages that are capable of suppressing acute inflammation and promoting fibroplasia (11–13).

T3 response reactions (EAACI hypersensitivity reactions type IVc) are mediated by type 17 T-helper cells, Tc17, ILC3, and other cells that produce IL-17 family cytokines, with the involvement of neutrophils, a hallmark of purulent inflammation. IL-17 regulates innate effectors and orchestrates local inflammation by inducing the release of pro-inflammatory cytokines and chemokines that can recruit neutrophils (with their potent production of defensins, hypochlorous acid, and other reactive oxygen/halogen species able to disinfect inflammatory foci) and enhance cytokine production by Th2 cells. Th17 memory cells acquire their phenotype when exposed to IL-6, IL-21, IL-23, and TGF-β provided by APCs. The main effector cytokines produced by Th17 cells are IL-17A, IL-17F, IL-21, IL-22, and granulocyte colony-stimulating factor. IL-17A and IL-17F are also produced by CD4+ and CD8+ T cells, T lymphocytes, and NK cells in response to IL-1β and IL-23. Their primary role is to generate protective immunity against fungi and extracellular bacteria. IL-17A and IL-17F activate ILC3 and stromal cells to produce IL-8, which also recruits neutrophils to sites of inflammation (5, 14–16).

The processes of suppression or downregulation of the immune response (immunosuppression) and the formation of immune memory should be considered as its mandatory final stages (1, 7). The mechanisms behind this downregulation are varied. They include the suppression of the proliferation and functions of T and B lymphocytes, leading to an increase in their rates of apoptosis. Additionally, they involve the activation of different types of suppressor cell clones around 22 known varieties, including T regulatory cells (Tregs), B regulatory cells (Bregs), mesenchymal stem cells, and myeloid-derived suppressor cells. There is also a shift in the cytokine profile, resulting in an imbalance between pro-inflammatory and anti-inflammatory activities. Furthermore, anti-idiotypic autoantibodies and autoantibodies targeting cytokines and their receptors can have clonally specific downregulatory effects, indicating that, to some extent, immunity is modulated by autoimmunity (17–19).

Immunosuppression can occur through various mechanisms: it may be physiological, such as during pregnancy or after a well-regulated immune response; natural, as seen in infancy and old age; or pathological, arising from conditions like parasitic infections, severe infectious diseases that provoke a strong immune response, congenital immunodeficiencies, or exposure to harmful external factors. Additionally, immunoexpressed states can frequently emerge as iatrogenic effects, particularly in the context of drug or radiation therapies. A lack of suppressive factors is linked to the breakdown of self-tolerance, leading to various autoimmune disorders. Conversely, excessive suppressive activity can contribute to the development of neoplastic and infectious diseases (20–23).

*T1-response reactions* (EAACI hypersensitivity reactions type IVa) are mediated by type 1 T-helper cells (Th1) and type 1 cytotoxic cells (Tc1), which develop their phenotype under the influence of IL-12, IL-23, and IFN-γ, produced by antigen-presenting cells (APC). Th1 cells produce large amounts of IFN-γ. Lymphotoxin and tumor necrosis factor-alpha (TNF-α), which are involved in disease pathogenesis due to their essential role in granuloma formation, the synthesis of IgG1 and IgG3 by plasma cells, and the ability to activate T-cell cytotoxicity. The T1-dependent immune response is enhanced by certain cells of innate immunity, including type 1 innate lymphoid cells (ILC1) and classically activated macrophages (MCP-1 or M1), but primarily by NK cells (24). Activated MCP-1 releases inflammatory mediators (reactive oxygen species (ROS), proteases, and pro-inflammatory cytokines, etc.), contributing to tissue damage at the site of antigen exposure. Tissue damage leads to clinical manifestations of hypersensitivity, which may vary depending on the specific antigens targeted (25–27).

The humoral part of the immune response involves different classes and subclasses of immunoglobulins. IgA is important in the mucosal surfaces, preventing pathogens from entering the body. IgA exists in two forms: monomeric (in serum) and dimeric (secreted by exocrine glands; Table 2).

**Table 2:** Humoral immunopathological responses are associated with systemic adaptive immunity reactions.

**Type of response**		**Cells and cytokines involved in the immune response**	**Pathological processes**	
			**Hyporesponsive**	**Hyperresponsive**
Mediated by immunoglobulins A		IgA antibodies	Recurrent sinus-pulmonary infections, diarrhea, allergic reactions, autoimmune pathology, dysbacteriosis	IgA nephropathy, celiac disease, Shenzhen-Genoha purpura
Immunoglobulin-mediated immunoglobulins G	IgG1	Ig G1 antibodies	Bacterial-viral infections, virus-induced bronchial asthma	Monoclonal gammopathy of undetermined significance, myeloma disease, paraproteinemia, lymphoproliferative diseases EAACI reaction type II
	IgG2	Ig G2 antibodies	Predisposition to infections with capsular microorganisms	
	IgG3	Ig G3 antibodies	Respiratory infections	
	IgG4	Ig G4 antibodies	Sinus-respiratory infections, recurrent pneumonia, and bronchiectasis	Autoimmune pancreatitis, Mikulicz syndrome, and other chronic inflammatory-fibrotic diseases
Immunoglobulin-mediated immunoglobulins M		Ig M antibodies	Primary and secondary immunodeficiencies. Condition after removal of the spleen	Acute and chronic infections, autoimmune diseases
Immunoglobulin-mediated immunoglobulins E		Ig E antibodies	Intestinal infestations	Hyperimmunoglobulinemia E syndrome; Job's syndrome EAACI reaction type I, worm infestations

*Immunoglobulin A* deficiency is diagnosed when serum IgA levels are <0.07 g/L (0.4375 μmol/L). It is the most common primary immunodeficiency. Many patients are asymptomatic, but some experience recurrent infections of the upper and lower respiratory tracts, sinuses, ears, as well as infectious and non-infectious diseases of the gastrointestinal tract (including celiac disease). This form of immunodeficiency can manifest as allergic reactions (such as bronchial asthma accompanied by adenoids) or as autoimmune disorders (such as inflammatory bowel disease, systemic lupus erythematosus, and chronic active hepatitis). IgA deficiency is often accompanied by various types of dysbiosis (28).

Several microorganisms (*Neisseria gonorrhoeae, Streptococcus pneumoniae, Haemophilus influenzae* type B, *Blastocystis*) can secrete enzymes that degrade IgA, leading to acquired IgA-associated immunodeficiency (29–31). IgA nephropathy, associated with IgA-containing renal immune complex deposits, vasculitis, and gluten-sensitive enteropathy, is accompanied by IgA excess.

Berger's disease, or IgA nephropathy, is a form of chronic glomerulonephritis characterized by the accumulation of IgA-containing immune complexes in the mesangium. The disease was first described by Jean Berger (1930–2011) in 1968. It is the most common form of glomerulonephritis worldwide. In young adults, it often presents with episodic hematuria, usually within a day or two after the onset of an upper respiratory tract infection. Older patients may present with asymptomatic microhematuria and proteinuria, which can only be detected by urinalysis. Renal failure may rarely develop (32, 33).

A systemic clinical manifestation of IgA-associated disorder, immunoglobulin A vasculitis (hemorrhagic vasculitis, Henoch-Schönlein purpura), is closely related to IgA nephropathy. It is a systemic vasculitis of small blood vessels that, in addition to the kidneys, affects the skin (purpura), joints (arthritis), and intestines (melena and abdominal pain) (34–36).

Gluten-sensitive enteropathy, a common genetic disease, the pathogenesis of which is based on sensitization to the prolamin proteins of some cereals, most frequently gliadin of wheat, and also secalin of rye, hordein of barley, etc. In the presence of genetic predisposition exists, typically associated with some HLA-DQ2 or HLA-DQ8 alleles, activation of T-cells and the production of antibodies (most often represented by IgA) to tissue transglutaminase and to endomysium (a wispy layer of areolar connective tissue that ensheathe individual smooth myocytes of intestine) occurs in response to the presentation of peptide epitopes of the above mentioned cereal prolamins.

Transglutaminase is involved because it modifies peptides of prolamins into a form that may stimulate the immune system more effectively, forming a complex with them prone to antigen presentation. Endomysium is altered due to the presence of surface transglutaminase. IL-15 is considered a key player in the pathogenesis of intestinal villi damage and atrophy in coeliac disease. Moreover, gliadin and possibly other prolamins may directly stimulate innate immune cells in a lectin-like manner; hence, the failure of the membrane digestion of these peptides can promote a disease (37). The entity is characterized by a wide range of versatile clinical manifestations and is often comorbid with other autoimmune diseases (38, 39).

*Immunoglobulin G* (IgG) is the most common class of antibodies in serum (comprising ~75%−80% of all immunoglobulins). It has the longest half-life blood circulation time (7–21 days) due to the function of the endothelial Fc-receptor of neonatal type (FcRn or Brambell's receptor), which establishes antibody recycling and protection from IgG endosomal degradation (1, 37). A similar receptor is responsible for the transmission of IgG across the placenta (38).

This class of immunoglobulins is capable of neutralizing toxins and viruses. By binding to the antigens of extracellular bacteria and fungi, it coats the surface of pathogens, facilitating their phagocytosis (a process known as opsonization), and also causes their immobilization and binding by agglutination. IgG-mediated opsonization allows pathogen recognition and uptake by professional phagocytes and elicits antibody-dependent cell-mediated cytotoxicity. In addition, IgG activates the classical pathway of the complement system. Some IgG antibodies against nuclear antigens can penetrate the nuclei of living cells and alter genetically determined processes (18, 40). In humans, there are four subclasses of IgG (about 65% belong to IgG1, 25% to IgG2, 6% to IgG3, and just 4% to IgG4. All the subclasses are characterized by different properties (Table 3).

**Table 3:** Characterization of immunoglobulin G subclasses.

**Subclass IgG**	**Plasma concentration (g/L)**	**Half-life**	**Fc-receptor binding to phagocytes**	**Complement activation**	**Penetration through the placenta**
1	3.82–9.28	21 days	High	High	Yes
2	2.41–7.00	21 days	Low	Middle	No
3	0.22–1.76	7 days	High	High	No
4	0.04–1.35	21 days	Middle	Not	Yes

It is considered that the immune response to most antigens involves a mixture of all four IgG subclasses; however, it creates difficulty in recognizing which of the subclasses is defective. Nevertheless, there are specific features that are distinctive for defects in different IgG subclasses. Attention should be paid to the temporal dynamics of immunoglobulin production. It is assumed that IgG3 and IgE are produced early after IgM, followed by IgG1 and IgG2. If the antigen persists, high-affinity IgG4 is produced, which, among other functions, has some anti-inflammatory potential and may facilitate fibroplasia (40).

The most frequent disorder related to immunoglobulins of class G is IgG1 deficiency (about 4%). Hypogammaglobulinemia is its main feature, because this subclass is normally the predominant one among IgG. Often, IgG1 deficiency is associated with deficiencies in IgA and IgM. Two groups of disorders dominate its clinical picture. The first is associated with bacterial-viral diseases of the respiratory tract, the second with virus-induced bronchial asthma (41, 42).

IgG2 is associated with the process of antibody formation toward polysaccharide capsular antigens of microorganisms. In this regard, IgG2 deficiency is associated with a predisposition to infections caused by encapsulated bacteria (*Neisseria meningitides, Streptococcus pneumonia*, and *Haemophilus influenza*). Clinically, it is manifested by otitis media, sinusitis, recurrent bronchitis, and chronic skin candidiasis. In severe cases, chronic obstructive pulmonary disease, pneumonia, and meningitis may develop. It has been established that IgG2 deficiency occurs in ~10% of patients with bronchiectasis. Selective IgG2 deficiency may be associated with Louis-Barr syndrome. A combined form of IgA and IgG2 immunodeficiency has been described. IgG2 deficiency is also associated with several autoimmune diseases (SLE, primary Sjögren's syndrome, juvenile diabetes mellitus, autoimmune hemocytopenia, and hemorrhagic vasculitis).

IgG3 is involved in the formation of specific immunity against bacteria colonizing the nasopharynx (*Moraxella catarrhalis* and *Streptococcus pyogenes*). IgG3 deficiency may present as selective immunodeficiency or in combination with IgG1 deficiency. Hypogammaglobulinemia is commonly diagnosed in such patients. These patients frequently exhibit hypogammaglobulinemia. IgG3 deficiency is associated with asthma, frequent exacerbations of chronic bronchitis, ENT pathologies, gastrointestinal infections, and herpetic infections.

Diagnosis of IgG4 deficiency is challenging, as this subclass of immunoglobulins may be present at very low concentrations in the serum of many healthy adults and all healthy children under the age of 10. Thus, there may not be enough evidence of antibody deficiency. Nevertheless, the combined deficiencies of IgG4 with IgA and IgG2 have been described, which manifest by sinus-respiratory infections, recurrent pneumonias, and bronchiectasis (43).

IgG4 excess is typical of a systemic hyper-IgG4 disease, a multi-organ illness of unknown etiology, characterized by chronic inflammation and fibrosis, most frequently altering the pancreas, tear and salivary glands, orbital fat, kidneys, retroperitoneal space, and thyroid gland. It occurs worldwide, but is most prevalent in the Japanese population. The local foci of the disease exhibit lymphocytic infiltration with IgG4-positive plasma cells and eosinophils, and dense fibrosis, sometimes with obliterating phlebitis (40, 41). At least 70% of patients have elevated serum IgG4 levels >1.4 g/L. If the IgG4 serum level exceeds twice the upper limit of normal, it is considered to be a pathognomonic hallmark of the disease (42).

Elevated IgG4 levels are also observed in several other diseases, such as pancreatic and biliary cancers, and may occasionally be seen in healthy individuals (43).

Quite often, primary and secondary immunodeficiencies of different Ig subclasses are combined, but at the same time, they are accompanied by sufficient concentrations of some other classes or subclasses of immunoglobulins. Thus, IgA deficiency is often associated with deficiencies in IgG2, IgG4, and IgG3; in such cases, these antibodies may be absent. Lack of all IgG (IgG1–IgG4 subclasses) is observed in common variable immunodeficiency. In Wiskott-Aldrich syndrome, deficiencies in IgG3 and IgG4 are most commonly identified. In ataxia-telangiectasia, IgG2 and IgG4 levels are reduced, and sometimes IgG3 deficiency is detected. In chronic candidiasis of the skin and mucous membranes, patients often have deficiencies in IgG2 and IgG4, or isolated deficiencies in IgG2 or IgG3. In HIV infection, there are decreased levels of IgG2 and IgG4, accompanied by increased levels of IgG1 and IgG3. Both radiation exposure and chemotherapy often cause decreased levels of IgG2 and IgG4 (7, 39, 40).

*Immunoglobulin M (IgM)* is the largest (pentamer consisting of five monomers), and it is the first antibody produced in the primary immune response to infection (known as acute phase antibodies).

Decreased IgM levels are observed in primary immune deficiencies (severe combined immune deficiency, agammaglobulinemia (Bruton's disease), congenital IgM deficiency), or secondary immunodepression (inflammatory diseases of the colon, nephrotic syndrome, burns, and other conditions associated with protein loss, exposure to immunosuppressants, cytostatic medicines, and radiation). It is also common for myeloma disease (of IgA- or IgG-types), for AIDS, and for the status after spleen removal. There may be a physiological decrease in IgM level typical in children aged 3–5 months (1).

Elevated IgM in the blood indicates an acute inflammatory process in acute and chronic bacterial, viral, fungal, and parasitic infections, autoimmune diseases (rheumatoid arthritis, systemic lupus erythematosus), in liver diseases (primary biliary cirrhosis, acute viral hepatitis), and multiple myeloma disease (of IgM-type). Immunoglobulin M levels can also increase after intense physical exercise, severe stress, when taking certain drugs (methylprednisolone, penicillamine, valproic acid, estrogens, oral contraceptives, antipsychotic and antiepileptic drugs, etc.) (28, 44).

*Immunoglobulin E (IgE)* is normally present at very low concentrations in the blood and plays a key role in the immune response to allergens and parasitic infections by releasing histamine and other inflammatory mediators through binding to mast cell and basophil surface receptors. It plays a crucial role in normal placentogenesis in pregnancy (45, 46). Increased IgE content is observed in atopic allergic diseases based on anaphylactic reactions (bronchial asthma, pollinosis, urticaria, atopic Quincke's edema, and atopic dermatitis, etc.). Sometimes it accompanies primary immunodeficiencies (hyperimmunoglobulinemia E syndrome, Wiskott-Aldrich syndrome), and may be observed in IgE-myeloma, as well as in worm infestations and worm larvae migration syndromes (10).

## 3 Local immunopathological reactions

*Type I interferonopathy* should be recognized as a separate immunopathological syndrome observed during local inflammation. Class I and III interferons are primarily the bioregulators of close-distance autocrine, juxtacrine, and/or paracrine action (autacoids), and play a key role in the immune defense of the organism against viral infections. In interferonopathies, their regulatory mechanisms are disturbed, resulting in either excessive or insufficient production of interferons (45, 46). Clinical signs of interferon insufficiency are tumor growth, recurrent acute and chronic viral and other intracellular infections. In the case of interferon hyperproduction, the clinical picture may include fever, skin manifestations, vasculopathies, interstitial lung damage, as well as involvement of the central nervous system and musculoskeletal system. The genetically determined interferonopathies are quite rare (1:100,000–1:10,000,000 individuals). Among them: STING-associated vasculopathy with onset in infancy (SAVI syndrome), STAT1 gain of function (GOF syndrome), proteasome-associated autoinflammatory syndrome (PRAAS), chronic atypical neutrophilic dermatosis with lipodystrophy and fever (CANDLE), monogenic systemic lupus erythematosus (SLE), spondyloenhondrodysplasia (SPENCD syndrome), adenosine deaminase-2 deficiency or autosomal recessive autoinflammatory disease DADA2, etc. Acquired interferon deficiency, which accompanies all acute viral infections and plays a crucial role in the outcome of the COVID-19 infection, is much more common (47–51). Interferon unresponsiveness in viral infections may result from anti-interferon autoantibodies (52, 53). Anti-interferon receptor antibodies can also exist and may either block or stimulate interferon receptors, developing as a result of an anti-idiotypic immune response (47, 48).

## 4 Systemic immunopathological reactions

Inflammation is always a local typical pathological process, a response of vascularized tissue to damage, driven mostly by the signals produced within its foci, and not by bioregulators that are external to them. This process is capable of creating barriers that prevent the systemic spread of pathogens and also limit the general action of bioregulators participating in focal events. The latter is known as the informational autochthony of the inflammatory focus. Preserving its local character, inflammation (together with neuroendocrine stress reaction proceeding in parallel) prevents the development of circulatory shock after injuries or infections. However, losing its locality due to the failure of barriers, it triggers or aggravates the systemic disorders, moving the body along the pathogenic pathway. The consequences of increasing systemic action of inflammatory mediators vary in severity, ranging from regular moderate acute-phase response and leukocytosis, which are the defensive systemic correlates of inflammation, to harmful shock-like states, including circulatory shock. These aspects were detailed in our recent article published elsewhere (7).

*Systemic inflammatory response syndrome (SIRS)* is a systemic correlate of inflammatory reaction in response to severe lesions of infectious and non-infectious nature, regardless of the localization of the inflammatory foci, provided their barrier function is insufficient.

The clinical term SIRS was initially used in 1987 by Cerra (49), to designate hypermetabolism and multi-organ failure in sepsis and toxic-septic shock. Later, in 1992, a consensus conference of the American College of Chest Physicians and the Society of Critical Care Medicine suggested the definition and set of SIRS criteria. These criteria include fever or hypothermia, tachypnea, tachycardia, and leukocytosis or leukopenia with a left shift in the leukocyte count (39, 40). SIRS is an “old wine in new wine skins”: a pre-shock condition or an extreme hyperergic variant of acute phase response, a typical pathological process well-known in pathophysiology since the 1950s (7).

As we noted above, local inflammatory reactions are primarily aimed at the localization and elimination of the damaging factor (e.g., primarily infection) with subsequent reparative regeneration. This is achieved by the production of various inflammatory mediators by epithelial cells, endothelial cells, mast cells, macrophages, and granulocytes, which in turn activate hemostasis and complement systems. Cytokines represent the most extensively studied group of inflammatory mediators. The spread of small amounts of cytokines from the foci of inflammation into the systemic bloodstream in normergic inflammation, which H. Bekemeier called “orthophlogosis,” is one of the main factors initiating other closely related systemic pathologic processes, such as stress, fever, and acute phase response (50). These systemic reactions support the local inflammatory process by enhancing leukocyte infiltration, increasing energy and substrate availability, and reinforcing the barrier function surrounding the affected area, thereby preserving homeostasis (7).

It should be noted that activation of both pro-inflammatory and anti-inflammatory immune reactions occurs in SIRS (51). In the case of a balanced interaction of pro-inflammatory and anti-inflammatory cytokines, the so-called *mixed antagonist response syndrome (MARS)* is formed. If one type of cytokine strongly predominates, a hyperactive systemic inflammatory response is formed, or a compensatory anti-inflammatory response (CARS-compensatory anti-inflammatory response) with immunosuppression prevails (52). These processes can evolve in a phased manner, for example, in cases of severe polytrauma, extensive burns, or septic conditions, where the volume and spread of the inflammation exceed the capacity of the barrier systems (7, 53). Based on clinical parameters, two of these varieties can already be distinguished.

Recently, in addition to the general concept of “systemic inflammatory response,” other close definitions and a variety of names for the development of the excessive systemic response of the innate immune system have been coined. These include “cytokine storm,” “cytokine tempest,” “secondary hemophagocytic lymphohistiocytosis (HLH) syndrome,” “macrophage activation syndrome (MAS),” and “virus-induced hyperinflammatory syndrome.” All these conditions are characterized by uncontrolled and excessive release of pro-inflammatory cytokines (and other inflammatory autacoids) into the systemic bloodstream. These cytokines disrupt blood rheology, induce endothelial expression of adhesion molecules, and impair tissue perfusion far beyond the original site of inflammation, eventually leading to multi-organ failure and death. The diversity of terms often reflects varying degrees of severity, predominant symptoms, or the conventions of specific medical disciplines or national schools (7).

Cytokine dysregulation often leads to systemic disorders of innate immunity, involving activation of macrophages, dendritic cells, and natural killer (NK) cells. A severe form of this dysregulation is observed in histiocytic disorders, marked by clinical signs of excessive systemic cytokine activity, including hyperferritinemia, coagulopathies, and pathological hemophagocytosis, where macrophages engulf blood cells and their precursors in the bone marrow, spleen, liver, and other tissues. This condition is known as hemophagocytic lymphohistiocytosis (HLH).

This phenomenon is known as *Hemophagocytic lymphohistiocytosis syndrome* (HLH, Hemophagocytic lymphohistiocytosis). Hemophagocytic lymphohistiocytosis can be either primary (genetically determined) or secondary, associated with certain diseases or immunodepressive states (both physiological and pathological, or even iatrogenic ones).

Primary HLH includes familial HLH and other congenital immunodeficiencies, such as Chediak-Higashi syndrome, Griscelli syndrome, X-linked lymphoproliferative syndrome, Wiskott-Aldrich syndrome, severe combined immunodeficiency, and Hermansky-Pudlak syndrome.

Secondary or reactive hemophagocytic syndrome is usually classified by the presence of a specific trigger. Its common triggers are infections, including viruses (primarily DNA-containing and also HIV or dengue fever viruses), bacteria (such as *Mycobacteria* and spirochetes), fungi (histoplasmosis), parasites (malaria, leishmaniasis), as well as autoimmune diseases and malignancies (especially lymphomas). Chronic infection caused by Epstein-Barr virus (EBV). It is a well-known trigger of HLH, especially in individuals with primary or secondary immunodeficiencies or malignancies (54).

The pathogenesis of HLH is linked to the impaired cytotoxicity of NK cells and cytotoxic T lymphocytes. In primary HLH, this is due to pathological mutations in granule-dependent cytotoxicity (mutations in the genes PRF1, SH2D1A, BIRC4, ITK, UNC13D, STX11, RAB27A, STXBP2, LYST, CD27, and MAGT1). In secondary HLH, also called macrophage activation syndrome (MAS, Macrophage activation syndrome) or more precisely macrophage activation-like syndrome (MALS), it is believed that hyperproduction of cytokines [specifically, IL-1β, interferon-γ (INF-γ), tumor necrosis factor alpha, soluble IL-2 receptor (CD25), IL-12] leads to the depletion of NK cells and cytotoxic T lymphocytes, accompanied by the activation of tissue macrophages that produce pro-inflammatory cytokines (IL-1, IL-6, IL-10 and IL-18). This explains the clinical and laboratory manifestations of the disease (fever, hypofibrinogenemia, hypertriglyceridemia, hyperferritinemia, hemophagocytosis, edema, and CNS damage) (55, 56).

The pathogenesis of HLH depends on a vicious circle: not only Toll-like receptors of innate immunity cells (primarily stimulated by pathogen-associated molecular patterns), but also their receptors for IL-1 and IL-18 (which are stimulated by excess levels of these interleukins) can, at the post-receptor level, activate the same intracellular instruments (myeloid differentiation primary-response protein 88 (MyD88), and IL-1R-associated kinase (IRAK) proteins). This leads to the continuous assembly and activation of the stimulating myddosome and inflammasome, resulting in a further increase in interleukin production by those cells (57).

Disrupted cytotoxicity also impairs the apoptosis of tumor- or virus-infected cells, triggering the release of compensatory IFN-γ and GM-CSF. These, in turn, stimulate macrophages, causing their uncontrolled activation, phagocytosis of blood cells, and pro-inflammatory cytokine release. CD163, a scavenger receptor on macrophages, serves as a key marker of their activation. CD163 binds to the haptoglobin–hemoglobin complex, activating heme oxygenase, which degrades heme into biliverdin, carbon monoxide, and iron. The latter is sequestered by ferritin, leading to hyperferritinemia, which promotes ROS production, oxidative stress, and tissue damage (54). This process underlies the synonym for HLH macrophage activation syndrome (MAS).

Sometimes it is much more difficult to differentiate MAS from cytokine dysregulation and balanced systemic correlate of inflammation (acute-phase response). Although SIRS rarely meets MAS criteria, excessive macrophage activation in sepsis may resemble MAS in terms of clinical, laboratory, and morphological characteristics (Table 4).

**Table 4:** Differential diagnosis of syndromes associated with systemic innate immune response.

**Indicator**	**Systemic inflammatory response syndrome**	**Cytokine dysregulation syndrome**	**Macrophage activation syndrome**
Hepatomegaly/splenomegaly/lymphadenopathy	–	+	+++
CNS dysfunction	–	++	++
Hemostasis disorder	–	++	+++
Multiple organ failure	–	+++	++
Cytopenia with involvement of more than 2 cell lineages	+/–	+	+++
Decreased or absent NK-cell and CTL activity	–	+/–	+++
Thrombocytopenia	–	+/–	++
Increased ferritin	+/–	++	+++
Elevated LDH, AST, ALT	+/–	++	+++
Hypertriglyceridaemia	–	+	+++
Hemophagocytosis in biopsy specimens	–	+	+++

Another entity associated with macrophage dysfunction is the syndrome associated with an excessive amount of circulating immune complexes (CICs). In immune complex diseases, excessive amounts of CICs are formed and not properly utilized by phagocytes. The normal clearance of CICs is disturbed, and the solubility of the formed immune complexes may be reduced. It leads to their deposition in the vascular wall and the development of inflammation (vasculitides) (56). Thus, CICs are deposited in the wall of microcirculatory vessels, mostly in the areas where blood pressure is relatively high and capillaries are tortuous (kidney glomeruli, retinal and joint vasculature, *plexus chorioideus*, etc.). The relatively low velocity of blood flow and low local temperature of the skin facilitate CIC-dependent vasculitis in the dermis. This process activates complement pathways, leading to the production of anaphylatoxins and cytokines, which subsequently attract leukocytes to the walls of small vessels in the skin. According to the EAACI classification of allergic diseases and hypersensitivity reactions, this syndrome is classified as a type III immune complex-mediated reaction.

The complement system is also directly related to the formation of immune complexes. About 50 proteins and peptides (~10% of blood globulins) are related to the complement system. These components normally circulate in an inactive form (except for D-factor and C3, which exist in plasma in minor quantities of active forms prevented from acting on self-cells by their ubiquitous surface inhibitor (C_inh_).

The complement factors are capable of self-assembling in response to certain immunological and non-immunological signals. In this process, they act as serine proteases and/or mutually recognizing receptors, and their short cleavage fragments work as pro-inflammatory peptide mediators. Hence, the complement system includes pattern recognition molecules, proteases, cell surface regulators and receptors, inhibitors, and other protein components. Most complement proteins are predominantly produced in the liver, secreted into the plasma, and from there transferred to extrahepatic tissues and organs. The reason is that there are some loci with restricted blood serum penetration; many cells of the immune system (especially macrophages and dendritic cells, as well as NK and some granulocytes) are capable of producing the complement components locally in extrahepatic areas. This is of great significance for close-distance focal paracrine effects of complement proteins. Moreover, like antibodies, complement is also able to act not only between the cells, but within the intracellular space (58). Complement is a crucial component of humoral innate immunity. Besides neutralizing microorganisms in cooperation with other systems, complement opsonizes and thus eliminates immune complexes, cellular debris, and apoptotic bodies. It also promotes the normal development of tissues and organs, stimulates regeneration, and participates in the switching of immunoglobulin subclasses (58–63).

The complement cascade is activated through three distinct pathways: the classical, lectin, and alternative. The classical pathway is initiated by the formation of an antigen-antibody complex. In the alternative pathway, foreign substances, pathogens, or damaged cells can bind directly to the C3b component, whereas the lectin pathway involves binding to mannose-binding lectin (MBL). All three pathways converge at the third complement component (C3), leading to the formation of additional effector components. The proteins C5, C6, C7, C8, and C9 assemble to form the membrane attack complex (MAC), which disrupts the membranes of pathogens or antibody-coated cells. Opsonization by C3b stimulates phagocytosis, and anaphylatoxins C3a and C5a attract macrophages and neutrophils, which release lysosomal enzymes and free radicals, thereby causing secondary tissue damage in the pathogenesis of inflammation (64–67).

**Figure 1:**
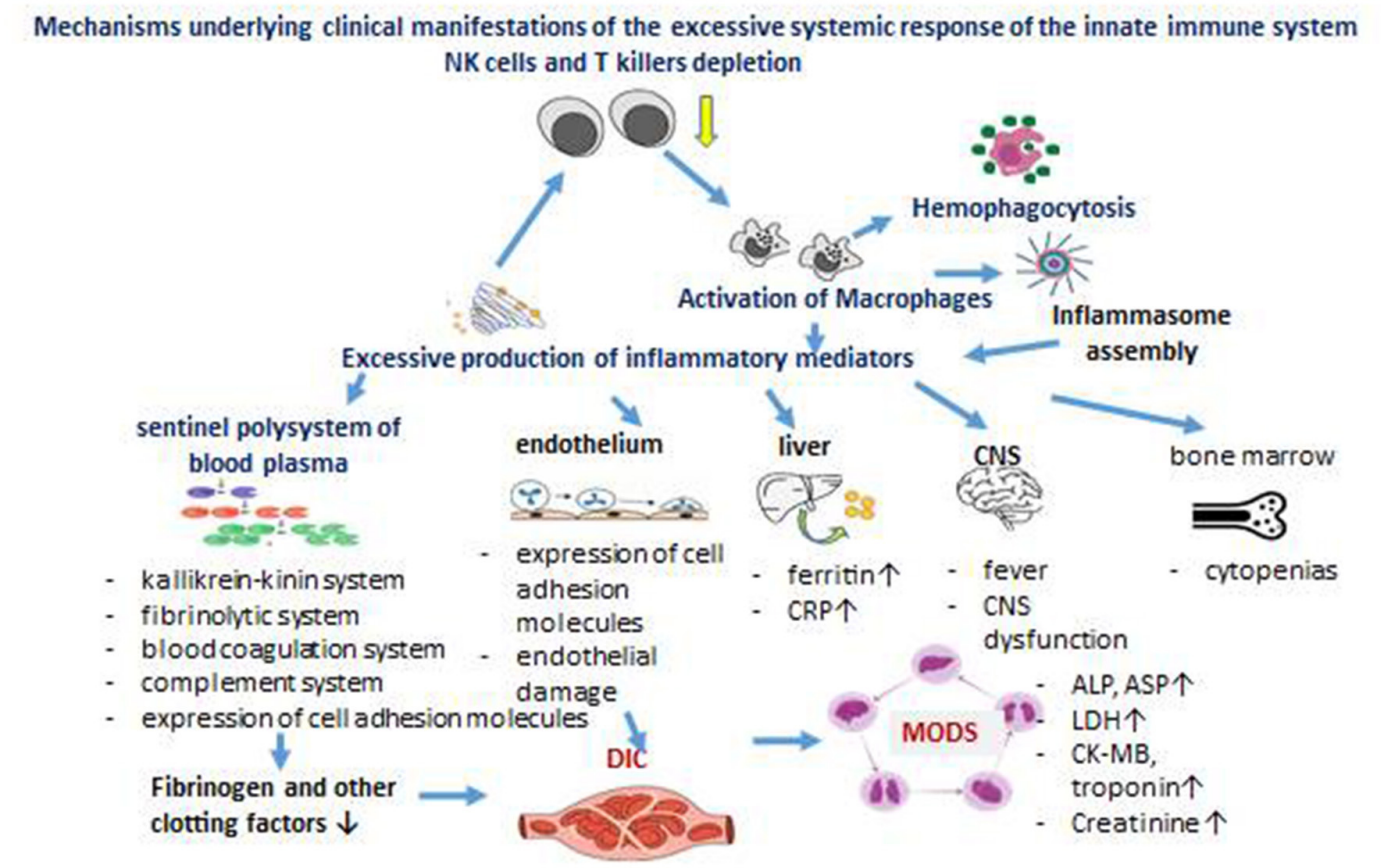
Mechanisms underlying clinical manifestations of the excessive systemic response of the innate immune system in SIRS.

Therefore, based on literature data and our studies, we have identified some clinical manifestations of chronic SIRS, which are presented in Figure 1.

The reduction of natural killer (NK) cells and cytotoxic T lymphocytes, whether primary or secondary, leads to the activation of tissue macrophages. These macrophages release pro-inflammatory cytokines, which further impair the functionality of NK cells and cytotoxic T lymphocytes. These inflammatory mediators initiate the activation of several interconnected blood plasma protein systems, including the kallikrein-kinin, fibrinolytic, coagulation, and complement systems, which can potentially result in disseminated intravascular coagulation (DIC). The systemic effects of cytokines impact the endothelium, liver, central nervous system (CNS), and bone marrow. Widespread endothelial damage, accompanied by the expression of adhesion molecules on endothelial cells and the depletion of clotting factors due to systemic coagulation activation, contributes to the onset of DIC. Multiple organ dysfunction syndrome (MODS), characterized by the progressive failure of two or more organ systems, is a likely outcome of DIC.

The complement system also interacts with many factors and systems in the body. It is tightly coordinated with other systems based on step-wise proteolysis (kinins, fibrinolysis, and blood coagulation) and has common activators with them. Their integrated functioning is sometimes mentioned as the sentinel polysystem of blood plasma (58). Moreover, complement coordinates with the clotting system. For example, thrombin cleaves and activates C5 to anaphylatoxin (C5a). Activation of the coagulation cascade (a stage of the hemostasis system aimed at fibrin production) can be considered not only as a process of preventing and stopping bleeding, but also as an emergency method to restore tissue integrity (as regards to epithelia, including endothelium, and to integument) by preventing the penetration of infection, while working in cooperation with the innate immune system. Such a commonwealth of complement and fibrin systems results in immunothrombosis.

### 4.1 Immunothrombosis

One of the most striking and life-threatening manifestations of an immune response is immunothrombosis. The concept and term were introduced in 2013 by Bernd Engelmann (Institut für Laboratoriumsmedizin) and Steffen Massberg (Medizinische Klinik und Poliklinik, Klinikum der Universität, Ludwig-Maximilians-Universität, Munich, Germany), who wrote: “Here, we summarize recent work suggesting that thrombosis under certain circumstances has a major physiological role in immune defense, and we introduce the term immunothrombosis to describe this process” (68).

Immunothrombosis is a reaction of the innate immune response that involves the formation of blood clots, primarily in microvessels. It facilitates the recognition, containment, and destruction of pathogens, thereby protecting the integrity of the body without causing large-scale secondary alterations. However, under various pathological situations, with excessive, abnormal activation of coagulation, it may lead to disseminated intravascular coagulation (DIC) and other thrombotic complications. Thrombosis associated with DIC is one of the leading causes of death worldwide (69–71).

The interaction between the immune and coagulation systems is particularly pronounced in viral and bacterial infections, as well as in autoimmune diseases. When pathogens enter the bloodstream, they trigger a variety of immune defense mechanisms, which in turn activate the coagulation cascade as a strategy to limit the dissemination of pathogen. The primary cellular effectors in this process are cells of innate immunity, especially neutrophils. While eosinophils and monocytes also express tissue factor (TF), their role in immunothrombosis has only been elucidated in recent years.

At the molecular level, immunothrombosis is governed by a complex interplay between the coagulation cascade, the complement system, and cytokines. These components establish multiple positive feedback loops, eventually leading to vascular occlusion when protective anticoagulant mechanisms, such as DNase I or activated protein C, are overwhelmed. Neutrophil extracellular traps (NETs), formed by neutrophils, eosinophils, and monocytes, and primarily aimed at pathogen elimination, also contribute to this process (72, 73).

A well-documented example of immunothrombosis is COVID-19. SARS-CoV-2 can induce arterial and venous thrombosis, a consequence of virus-induced immune dysregulation (cytokine storm). The pathophysiological reactions to SARS-CoV-2 include endothelial dysfunction, complement activation with the formation of C3a and C5a, cell lysis, a cytokine storm, NET formation, TF release, coagulation cascade activation, elevated PAI-1 secretion from mast cells and basophils, and suppression of fibrinolysis (74). These processes contribute to microvascular thrombosis, especially in the pulmonary circulation (75).

Notably, immunothrombosis in COVID-19 differs significantly from both overt DIC and sepsis-induced coagulopathy (SIC). Laboratory findings include elevated fibrinogen levels, significant increases in D-dimer, absence of bleeding, and predominance of microthrombosis, particularly in the lung microvasculature (76).

However, the pathogenesis of COVID-19-related thrombosis is multifactorial. Some authors argue that COVID-19-associated coagulopathy does not meet the criteria for classical thrombotic microangiopathy but rather exhibits features of complement-mediated endothelial injury and von Willebrand factor dysregulation (77).

The severity of COVID-19 correlates with markers of cellular and humoral immunity. Pronounced leukopenia, lymphopenia, elevated lactate dehydrogenase, and procalcitonin levels are more commonly observed in patients with fatal outcomes (78). Interleukin-6 has emerged as a key prognostic cytokine, demonstrating predictive value at the time of hospital admission.

The interplay between immune and hemostatic systems is also mediated by microvesicular interactions involving blood cells and the endothelium. These microparticles, enriched with membrane proteins, organelle contents, and cytoplasmic elements, serve as potent signaling platforms (79). In COVID-19 patients, microparticle levels from various sources vary significantly between admission and discharge, with a decline observed in patients with favorable outcomes.

Similar mechanisms are observed in autoimmune conditions such as antiphospholipid syndrome (APS), which is characterized by arterial and venous thrombosis and pregnancy complications due to microvascular injury. Contributing mechanisms in APS include upregulated TF expression, adhesion molecule activation, complement-mediated endothelial damage via MAC, and the chemoattractant function of C5a. Monocytes release TF, cytokines (e.g., TNF-α, interleukins, type I interferons), and microparticles. Neutrophils produce reactive oxygen species and NETs. Persistent endothelial activation by antiphospholipid antibodies may result in progressive occlusive vasculopathy (80).

Thus, immunothrombosis can be considered a mechanism of thrombosis that develops against the background of dysregulation of cellular interaction in diseases. Its pathogenesis involves the activation of immune defense against pathogens in the bloodstream and autoimmune processes.

The key trigger of immunothrombosis is the inflammasome. Proteolysis in it (during pyroptosis) results in the release of tissue factor, which forms a complex with clotting factor VII to activate factors IX and X, generating thrombin and leading to fibrin formation and platelet activation. Furthermore, mechanisms of immunothrombosis formation have been described, involving the STING (stimulator of interferon response) and HMGB1 (high-mobility group protein B1 or amphotericin B) proteins (81, 82).

Clinically, immunothrombosis is manifested by the classic DIC syndrome, which can be classified into three types. The first type of DIC, characterized by suppressed fibrinolysis, is often observed in sepsis and is associated with multi-organ failure. It is characterized by an abnormal increase in coagulation activity. In the second type of DIC, fibrinolysis is abnormally activated, and sometimes fatal bleeding may follow. The third type of DIC is an intermediate one, characterized by a balanced fibrinolysis. To treat the clinical symptoms of DIC syndrome, the therapeutic intervention should be performed according to these three types (83).

However, all these manifestations are related to the acute phase of the disease. If acute inflammatory reactions do not stop (for example, due to persistence of non-eliminated pathogen or non-infectious phlogogenic agent, like uric acid salt crystals in gout), chronic systemic correlate of inflammation (SCI, *systemic chronic inflammation*) forms, which can cause secondary damage to tissues and organs and lead to chronic critical illnesses. SCI is commonly mild, often goes unnoticed, and can last for months or even years. The usual classic local signs of inflammation redness, swelling, local heat, and pain, are virtually absent. Instead, psycho-emotional and cognitive changes, as well as immune-related dysfunctions confirmed by laboratory tests, become more prominent. Uniform criteria for SCI have not yet been defined. (19, 78, 79). Immunopathological syndromes associated with adaptive immunity serve as evidence of the importance of a balanced immune system. Inappropriate activation, deficiency, or overreaction of various elements of the adaptive immune system can lead to serious diseases. Moreover, the equilibrium between local and systemic defensive reactions, as well as the delineation of their respective spheres of activity, are essential; otherwise, they will come into conflict and mutually emasculate their defensive potential, increasing the costs of defense and promoting self-harm (7).

**Figure 2:**
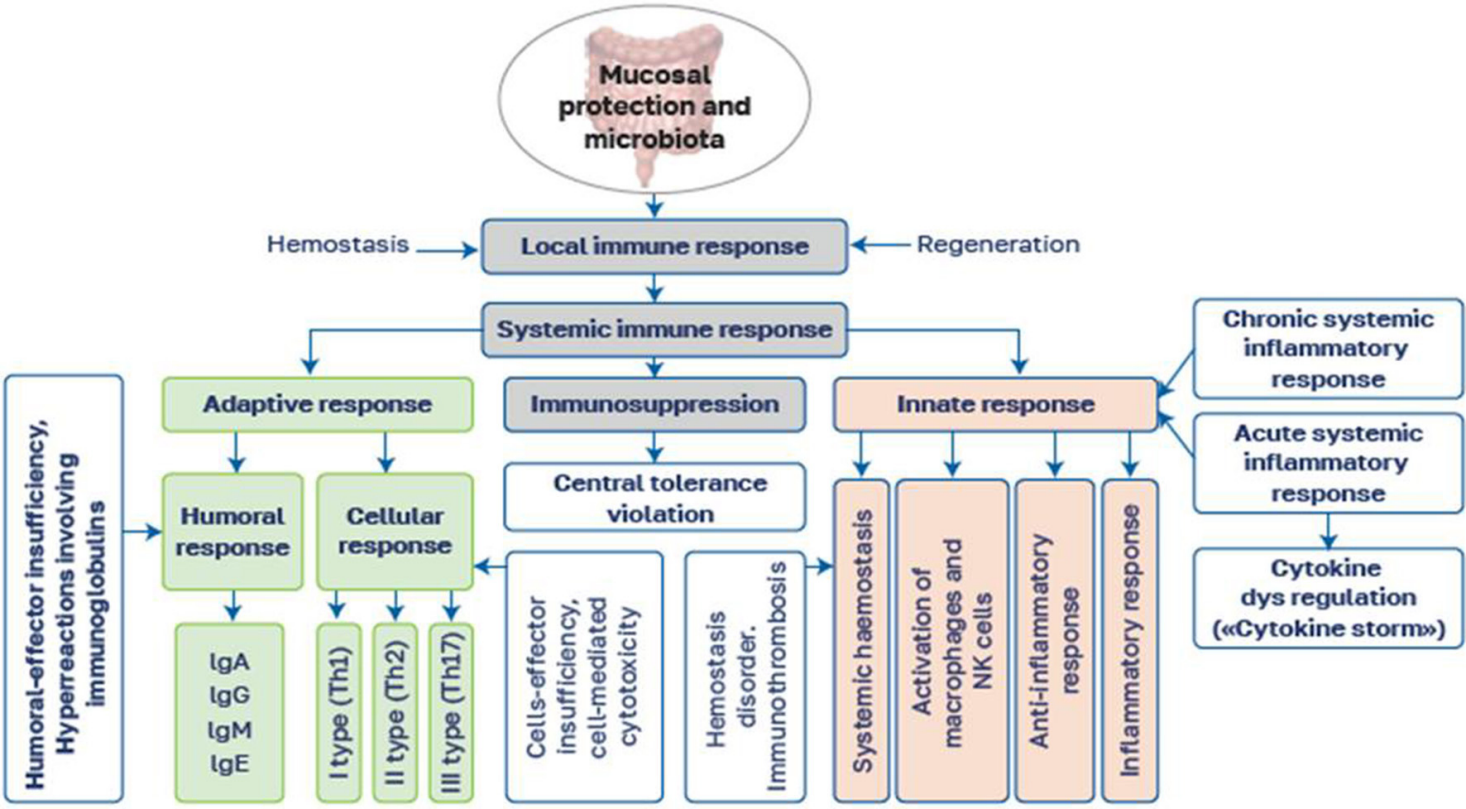
Types of immune response disorders under the influence of various factors.

The general pattern of immune response disorders is shown in Figure 2.

## 5 Conclusion

Innate immunity is the first line of defense of the organism against pathogens and damage, and maintains homeostasis. The mechanisms of innate immunity are primarily provided by localized responses (83–86). Inflammation is aimed at the localization, dilution, isolation, and elimination of the agent that caused the damage, as well as the restoration of damaged tissue.

At this stage, it is crucial to distinguish among various pathological conditions, such as dysregulated local inflammation, type I interferonopathies, and degenerative disorders. Local inflammation never occurs in isolation; it is invariably associated with a complex interplay of systemic processes. The systemic release of inflammatory mediators (autacoids) can give rise to broader physiological effects, including the acute-phase response, fever, leukocytosis, and stress-related responses.

The outcome of these interactions depends on several factors: the severity and extent of inflammation, the effectiveness of barrier functions, the anti-inflammatory actions of stress hormones, and the virulence or pathogenicity of the initiating agent. These factors collectively determine the nature and intensity of the systemic inflammatory response.

A balanced acute phase response, aligned with normergic inflammation, generally represents an adequate and proportionate reaction to infection or injury. In contrast, an uncontrolled systemic response, typically following the failure of local containment mechanisms, results in a pathological surge of cytokines, dysregulation of the component system, macrophage hyperactivation, and immunothrombosis. These phenomena are characteristic of systemic inflammatory syndromes and represent critical points for clinical intervention. Timely recognition of such conditions offers new opportunities for diagnosing and treating immune-mediated disorders.

It should be noted that in primary or secondary immunodeficiency disorders, adaptive immunity does not function properly, which leads primarily to the development of various infections (87–89). In case of the pathogen persistence, chronic inflammation occurs. Disturbances in the mechanisms of immune surveillance led to tumor growth.

Autoimmune diseases arise from inappropriate autoreactivity, whereas allergic disorders result from excessive, misdirected, or dysregulated immune responses to harmless antigens. These responses, while originating from the adaptive immune system, may cause more harm than protection.

A comprehensive understanding of these immunopathological syndromes, including the mechanisms underlying their onset and progression, is essential for the development of more precise diagnostic tools and targeted therapeutic strategies.
